# Canadian Sex Workers Weigh the Costs and Benefits of Disclosing Their Occupational Status to Health Providers

**DOI:** 10.1007/s13178-018-0339-8

**Published:** 2018-05-21

**Authors:** Cecilia Benoit, Michaela Smith, Mikael Jansson, Samantha Magnus, Renay Maurice, Jackson Flagg, Dan Reist

**Affiliations:** 1 Canadian Institute for Substance Use Research and Department of Sociology, University of Victoria, 2300 McKenzie Ave., Victoria, BC V8N 5M8 Canada; 2 Canadian Institute for Substance Use Research, University of Victoria, Victoria, BC Canada; 3 Department of Sociology, University of Victoria, Victoria, BC Canada

**Keywords:** Sex work, Unmet health care needs, Stigma, Disclosure, Health care encounters, Agency

## Abstract

Prostitution stigma has been shown to negatively affect the work, personal lives, and health of sex workers. Research also shows that sex workers have much higher unmet health care needs than the general population. Less is known about how stigma obstructs their health-seeking behaviors. For our thematic analysis, we explored Canadian sex workers’ accounts (*N* = 218) of accessing health care services for work-related health concerns. Results show that participants had mixed feelings about revealing their work status in health care encounters. Those who decided not to disclose were fearful of negative treatment or expressed confidentiality concerns or lack of relevancy. Those who divulged their occupational status to a health provider mainly described benefits, including nonjudgment, relationship building, and comprehensive care, while a minority experienced costs that included judgment, stigma, and inappropriate health care. Overall, health professionals in Canada appear to be doing a good job relating to sex workers who come forward for care. There is still a need for some providers to learn how to better converse with, diagnose, and care for people in sex work jobs that take into account the heavy costs associated with prostitution stigma.

## Introduction

Erving Goffman (1963) defined stigma as a social attribute or mark that separates those of us considered to be “normal” from “others,” based on dominant cultural stereotypes. Goffman (1963) noted some stigmatized traits or behaviors that are usually concealable from others, making those who bear them “discreditable” rather than outrightly “discredited.” For the discredited, their stigmatized statuses (race, body weight, physical ability, etc.) are visibly conspicuous, making concealment impossible. The discreditable, however, can hide their tainted characteristics or conditions (e.g., disability, sexual orientation, minority gender, or occupational status). This leads some to withhold disclosure, including in health care settings, unless they are in situations where the benefits of coming out are perceived to outweigh the risks or costs of stigmatization (Corrigan & Matthews, 2003; Link & Phelan, 2014). Stigmatized individuals thus carefully weigh the risks and benefits of disclosure (Broekema & Weber, 2017) and practice what Gronholm, Thornicroft, Laurens, and Evans-Lacko (2016) refer to as “conditional disclosure” that is dependent on past experiences and current circumstances. As Ragins (2008) notes, divulging or not divulging is preceded by an individual’s “assessment of the risks, benefits, and perceived consequences of disclosure within an environmental context” (p. 198).

Individuals can experience benefits from disclosing their discreditable status to others, including more open communication and greater acceptance and normalization (Corrigan, Morris, Michaels, Rafacz, & Rüsch, 2012; Fisher & Akman, 2002). The motives of individuals and their previous experiences of disclosure also affect the process of deciding to reveal a potentially stigmatizing status (Chaudoir & Fisher, 2010). In some situations, individuals may feel empowered enough to actively resist tainted identities and refuse to accept the imposition of a stigmatizing master status (Benoit, Jansson, Smith, & Flagg, 2017; McCabe & Leas, 2008; Wahl, 1999; Watson, 2002). Other benefits include improved self-esteem and psychological well-being (Corrigan, Kosyluk, & Rüsch, 2013), stronger social support systems, greater job satisfaction (Day & Schoenrade, 1997), increased empathy and support (Eaton, Ohan, Stritzke, Courtauld, & Corrigan, 2017), enhanced capacity to access health and social services (Corrigan & Matthews, 2003), and greater health equity (Neal, Schrader, Hyndman, Boyce, Phillips, Smith, et al., 2014).

Sex work is a highly discreditable status in most countries (Benoit, Ngugi, Roth, Jansson, Hallgrimsdottir, & Sharpe, 2013; Benoit et al., 2017; Biradavolu, Blankenship, Jena, & Dhungana, 2012; Foley, 2017; Ngugi, Benoit, Hallgrimsdottir, Jansson, & Roth, 2012; Sanders, 2017; Vanwesenbeeck, 2001) that is associated with three types of “taint”: physical taint from contact with bodily fluids and bodies, social taint from engaging in servile work and being potentially associated with other stigmatized groups (such as sex work clients, substance users), and moral taint from having their work be perceived as “somewhat sinful or of dubious virtue” (Ashforth & Kreiner, 1999, p. 415). Sex work operates in many countries in criminalized environments (exceptions include the Netherlands, Germany, New Zealand, and some states in Australia) and workers tend to be shunned or pitied but seldom granted agency (Vanwesenbeeck, 2001). Sex workers across many geopolitical contexts whose occupation is known often recount inappropriate care from health care providers, including through disrespectful and abusive language, public humiliation, physical separation from other patients, inferior service, inflated charges for private health care services, outright denial of care, and blame when reporting sexual assault (Aral, St. Lawrence, Tikhonova, Safarova, Parker, Shakarishvili, & Ryan, 2003; Foley, 2017; Ghimire, Smith, & van Teijlingen, 2011; Gorry, Roen, & Reilly, 2010; Ngo, Ratliff, McCurdy, Ross, Markham, & Pham, 2007; Phrasisombath, Thomsen, Sychareun, & Faxelid, 2012; Porras, Sabido, Fernandez-Davila, Fernandez, Batres, & Casabona, 2008; Scorgie et al., 2013; Sprankle, Bloomquist, Butcher, Gleason, & Schaefer, 2017; Stadler & Delaney, 2006). It is within these types of potentially stigmatizing health care settings where only about 10% of the participants in Canada and the UK—both countries with public health care systems—had disclosed their involvement in sex work to health professionals (Bungay, Kolar, Thindal, Remple, Johnston, & Ogilvie, 2013; Jeal & Salisbury, 2007). Sex workers contending with other stigmatizing identities, such as minority gender (Socías, Marshall, Arístegui, Romero, Cahn, Kerr, & Sued, 2014) and illicit substance use (Benoit, McCarthy, & Jansson, 2015a), were especially reluctant to reveal their occupational status to health care providers. In the case of these sex workers, a “web of stigmatization” often results (Wailoo, 2006), wherein the stigma associated with sex work is linked to or intersects with other stigmatizing statuses and becomes manifested in a diverse range of social contexts, including in the health care system where individuals may face a variety of obvious and more subtle barriers to having their health care needs met (Benoit et al., 2017; Hankivsky, Reid, Cormier, Varcoe, Clark, Benoit, & Brotman, 2010; Earnshaw, Smith, Cunningham, & Copenhaver, 2015; Hankivsky & Christoffersen, 2008; Orchard, Farr, Macphail, Wender, & Young, 2012).

Yet, there is a scarcity of research relating any benefits from “coming out” in health care settings for individuals engaged in sex work, such as those noted above for other discreditable groups (Corrigan et al., 2012; Fisher & Akman, 2002). Only two studies could be found linking benefits to post-disclosure of work status: Nguyen, Venne, Rodrigues, and Jacques (2008) found that study participants reported more comprehensive and continuous care from providers who portrayed a nonjudgmental attitude when their sex work was revealed, and Abel (2014) uncovered that disclosure to regular doctors led to more comprehensive check-ups for some study participants. There is thus a need for deeper understanding of the costs but also the benefits of disclosure and how sex workers, as agentic health care seekers, navigate divulging a potentially stigmatizing status in their specific socio-legal context.

### The Canadian Context

Canada is a liberal democracy that has taken an increasingly punitive approach to prostitution, while at the same time continuing to embrace universal health care for all residents. Canada’s recent laws related to prostitution have been driven more by ideology than empirical evidence, including the current dominant ideology that conflates sex work with sex trafficking (Jeffrey & Sullivan, 2009; van der Meulen, 2011). In 2014, the Canadian government under the Conservative Party enacted Bill C-36, which legislated the Protection of Communities and Exploited Persons Act (PCEPA). According to this new legal framework, sex workers may provide sexual services at fixed indoor locations; communicate with others for the purpose of offering or providing sexual services for consideration so long as this communication does not occur in a public place that is next to a school ground, playground, or day-care center; advertise their own sexual services; and pay for services with profits from the sale of their own sexual services (e.g., accounting, security) when that compensation is proportionate to the service offered (Department of Justice, 2014). While the Criminal Code amendments enacted in PCEPA effectively permit many sex work-related activities, they make it illegal for clients to obtain sexual services in any venue or to communicate in any place—public or private—for the purpose of obtaining sexual services for consideration. Moreover, it is now illegal for newspaper/magazine publishers, website administrators, and web-hosting services to publish advertisements for any sexual services (Department of Justice, 2014). This legal framework implies that, in theory, sex workers should not be barred from disclosing their work status out of fear of being criminalized, yet in practice, they may be reluctant to do so for fear of criminalizing their clients and others in their social networks.

At the same time, Canada’s nearly half-century old public health care system—called Medicare—is framed within a human rights approach that aims for fairness and equity for residents by guaranteeing access to needed health care irrespective of social position or personal circumstances. The national system is comprised of health insurance plans that provide coverage to all citizens and landed immigrants residing in the country’s provinces and territories. The system is publicly funded and administered on a provincial or territorial basis, within overarching regulations established by the federal government.

As with other countries impacted in recent decades by neoliberal economic and social policies, inequalities in access to health care persist in Canada, despite a robust health care system. Nearly 15% of Canadians report unmet health care needs, indicating that social inequalities in the country continue to affect health outcomes; this is especially the case for marginalized and stigmatized groups, including the homeless, indigenous peoples (Pauly, MacKinnon, & Varcoe, 2009) and sex workers (Neal et al., 2014). In fact, the percentage of unmet health care needs for sex workers is nearly three times higher than the general population (Benoit, Ouellet, & Jansson, 2016). Sex workers also do comparatively poorly on most other social determinants, which we discuss further below.

This article aims to explore sex workers’ complex decision-making regarding whether or not to make known their work status to health providers and the consequences of their decisions for receiving appropriate health care. Our research question is under which conditions and for what reasons do sex workers disclose (or conceal) their occupational status to (from) health care providers and what are the outcomes of these decisions?

## Methods

### Research Design

The data for this analysis are part of a community-engaged research project conducted in 2012–2013 that collected in-person data capturing the perspectives and experiences of five groups directly or indirectly affected by the sex industry: (a) sex workers, (b) intimate partners of sex workers, (c) those who purchase sexual services, (d) those who manage commercial sexual exchanges, and (e) those who are involved in providing health and social services to sex workers or in implementing laws. Collaborators included sex worker-led organizations, other outreach agencies, and public health or human rights groups. Collaborators assisted in designing the multi-project study, helped with recruitment of the participants, and supported interpretation of the findings. The analysis for this paper represents the sex worker portion of the project, which comprises questionnaire and interview data collected from 218 adult sex workers in Canada.

Our qualitative analysis is based on the detailed accounts elicited during the interview portion of the face-to-face meeting where participants were asked: Have you accessed health care services for (sex) work-related health concerns? Several probes were asked to further elicit to what extent and under what conditions participants experienced unmet health needs, stigma, and discrimination, as well as how participants responded to both positive and negative health care experiences. The benefits and costs of disclosing sex work or other stigmatizing statuses was not directly asked, though, as reported below, this was a major theme brought up independently by most participants when describing their health care experiences. Fourteen missing transcripts (occurred because of technical problems, participants not wishing to be recorded, or the participants only completing a portion of the interview) brought the number of transcripts for qualitative analysis to 204. Interviews conducted in French were translated into English before analysis.

### Recruitment and Sampling

The recruitment criteria for sex worker participation in the study included being aged 19 years or older, being legally able to work in Canada, and having delivered a minimum of 15 sexual services to clients in the past 12 months. Sexual services were considered to include, necessarily but not exclusively, direct physical contact between a sex worker and a client. We developed these criteria in collaboration with our community partners over the course of the development of the research project. These criteria were designed to recruit participants who were the age of majority in all jurisdictions and thus could potentially be subjected to criminal charges related to prostitution, who were habitually or regularly engaging in sex work, and who had direct physical contact with clients. All participants received an honorarium of $60 CAD for their participation. To avoid biasing the sample, we did not mention the monetary gift in our initial efforts to recruit participants. The honorarium was mentioned only after the participant had shown interest in the study.

Recruitment sites were six Canadian cities: St. John’s, Newfoundland; Montreal, Quebec; Kitchener-Waterloo-Cambridge, Ontario; Wood Buffalo (Fort McMurray), Alberta; Calgary, Alberta; and Victoria, British Columbia. These cities were selected from a sample of 93 potential cities because of diversity in regard to social and institutional factors such as their geographical variation, their difference in population size (i.e., medium and large cities), education, income, and because the researchers had established strong partnerships with sex worker agencies and other support organizations in each of them. Funding limited including more cities in the study.

We used nonrandom purposive sampling, which means that the selection of the participants was based on a specific criterion (Cresswell, 2003): in this case, currently working in the sex industry in Canada. Purposive sampling was useful as we were able to find participants who shared an occupational category, thereby allowing for an investigation of themes across and within their responses (Ritchie & Lewis, 2003). Although this process involved deliberate choices, it did not mean that undue bias was involved in the choices that were made. Instead, participants were recruited using diverse strategies to ensure the final sample illustrated characteristics that allowed us to consider our phenomenon in greater detail (Neuman & Robson, 2009). We worked to overcome sampling bias by using multiple concurrent recruitment strategies in each research site; these strategies were used in our earlier studies related to sex workers and other marginalized groups (Benoit et al., 2013, 2014, 2015a, 2016; Benoit, Smith, Jansson, Magnus, Ouellet, Atchison, Casey et al., 2016; Benoit, Magnus, Phillips, & Marcellus, 2015; Benoit, McCarthy, & Jansson, 2015b; Marcellus, MacKinnon, Benoit, & Phillips, 2015; Ngugi et al., 2012). These strategies include direct phone and email contact with escorts advertising on web sites and directories advertised on the Internet; advertising the study in local newspapers, on participant-related websites, and in social support offices and health clinics; using respondent-driven sampling, and with the help of community partners, hiring former sex workers as experiential research assistants, i.e., individuals hired mainly on the basis of their interest and extensive personal experience as workers in the sex work sector (Benoit, Jansson, Millar, & Phillips, 2005, p. 271). We adjusted our strategies throughout to ensure that no one strategy became dominant, that is, that the sampling bias from a particular strategy would not greatly affect the overall sample.

In its final form, the instrument took an average of an hour and a half to administer, including both a questionnaire component (approximately 60 min) and a small number of follow-up questions (approximately 30 min) to better understand involvement in sex work in participants’ public and private lives. Questions ranged from their entry into the industry to their experiences with protective and health services. The narrative orientation in this part of the interview allowed the participants to describe their thoughts and experiences in greater detail than the questionnaire format permitted.

While conducting the interviews, it became clear that many participants had provided, both historically and concurrently, professional sexual services in various work settings. However, in order to identify broad trends across current work type, they were grouped into three categories: independent street-based work, independent indoor work, and managed indoor work. Participants were assigned to a “managed” category if they answered yes to the question “do you have a supervisor?” A supervisor was defined as “(a) a person who earns an income from providing direction to sex workers, including training, hiring, monitoring, disciplining, and setting workplace standards; and (b) a person who instructs, directs, and controls sex workers in the performance of their duties.” Participants were categorized as independent street-based if they had solicited on the street or delivered services outdoors (e.g., in a park or vehicle) once a week or more in the last 12 months, even if they also engaged in other work types. None of the street-based workers in the sample had supervisors at the time of the interview, though some reported they engaged in managed street-based work in years prior. Participants in the indoor-based categories delivered services in-call or out-call, i.e., at the residence or room belonging to the client, worker, or business/agency.

### Ethical Considerations

Sex workers, a hidden and hard-to-reach population, are cognizant of the harms of unethical research. Over the last 20 years, our research team has developed a community-engaged research approach that involves trust, confidentiality, mutual benefit, and long-term commitment with marginalized groups facing formidable health inequities (Benoit et al., 2005; Leadbeater, Banister, Benoit, Jansson, Marshall, & Riecken, 2006). Our research goes beyond examining individual risk factors that tend to blame people for their situation to reveal the underlying determinants of their health inequities and develop methodological and ethical solutions to arrive at valid and reliable findings. Our community-engaged participatory approach has also been applied to the study of how stigma affects groups and to developing sound ethical protocols to ensure confidentiality and anonymity of sex workers who come forward to talk about their treatment by police, health providers and members of the general public, among others. We have designed robust mixed methods studies that employed validated measures and detailed participant accounts, allowing for comparison to other populations and at the same time shedding light on in-group heterogeneity.

Ethics approval for this particular study was obtained from the University of Victoria, the lead author’s institution, after consulting with community partners to develop an appropriate proposal for the target population. Verbal informed consent was obtained from all participants and permission was granted for the use of audio-recording equipment during the interview. Participants were assured of their ability to end the interview at any time and the confidentiality of the data they shared with researchers. All audio recordings were transcribed and any identifying details redacted. All participants quoted below were given pseudonyms to protect anonymity while at the same time bringing intimacy to the personal accounts.

### Analytical Procedures

Our thematic analysis focuses on “the ways individuals make meaning of their experience, and, in turn, the ways the broader social context impinges on those meanings” (Braun & Clark, 2006, p. 81). This approach helps the researcher to identify, analyze, and relate patterns or themes within the rich descriptive data (Braun & Clark, 2006). Participants’ accounts were thus coded thematically using NVivo 10 software following Braun and Clark’s (2006) multi-step guidelines for conducting thematic analysis. Initial coding involved the second author reviewing all transcripts in order to gain familiarity with the data and then generating a preliminary coding scheme to describe the most salient themes. The first and other authors also independently developed preliminary coding schemes through analyzing a random subsample of the interview transcripts. The team of authors then compared coding schemes and through several steps of revisiting the data and comparing coding strategies and their applications achieved consensus on a final coding structure (Bradshaw & Stratford, 2010). Similar codes were grouped together into themes and subthemes in order to make sense of the data that was present in the transcripts. The second author applied this finalized coding structure to the entire set of transcripts and the first and fourth authors checked the final coding in NVivo before the findings were written up.

## Results

### Participants

A summary of some broad participant demographic data is provided in Table 1. Indigenous participants identified as First Nations, Metis, or Inuit. Sixty percent of the participants engaged primarily in independent indoor work, 19% in managed indoor work, and 21% engaged in independent street-based work. The number of years in the sex industry ranged from less than 1 to 34 years, with a median of 6.8 and mean of 9.7 years.

**Table 1:** Overview of sex workers’ characteristics

Adults in the sex industry (*n* = 218)	
*Characteristics*	
Gender	
Women	76%
Men	17%
Trans	7%
Age (mean)	34 years
Annual personal income (median)	$39,500
Ethnicity	
Visible minority	12%
Indigenous	19%
Other	69%
Married/common law	30%
Work location	
Indoor-based	79%
Street-based	21%

### Thematic Findings

A major unanticipated theme emerging from the qualitative analysis—brought up by 80% of the participants without a specific interviewer probe—involved the issue of whether or not to disclose their occupation to health providers. The remaining 20% of the participants either did not discuss disclosure in their accounts or answered that they had not accessed health care for work-related concerns and therefore did not recount any relevant encounters.

Among those who did share accounts and brought up disclosure (*n* = 161), a minority (37%) said they had never revealed their sex work to a health provider, while the majority (63%) reported doing so at least once (see Table 2). Next, we present participants’ accounts of why they chose not to divulge their occupation and followed by why they did as well as the benefits and costs for doing so.

**Table 2:** Prevalence of sex work disclosure in sex workers’ accounts of health care experiences in Canada (*n* = 204)

	*f*	%
Disclosure not stated or unclear or no work-related health encounters described	43	–
Did not disclose	59	(37 of 161)
Ever disclosed	102	(63 of 161)
Only benefits described	58	57
Only costs described	16	16
Costs and benefits described	27	26
Neither cost nor benefit described	1	1

#### Nondisclosure of Sex Work to Health Providers

The 37% (59 out of 161) of our participants who had not disclosed their occupation to health care providers recounted three main reasons for not doing so: (1) fear they would be judged and mistreated, (2) there was no need for the health provider to know, and (3) concern that their privacy would not be respected. Participants were also aware that there were costs of nondisclosure, including blocking access to appropriate care and weakening trust and honesty in the patient-provider relationship.

##### Anticipated Stigma

Fifty-three percent of the participants (31/59) who had not disclosed their occupation to health care providers reported that part of their decision to withhold that information was due to a fear of judgment from providers. Ava (independent indoor, Calgary) put it this way: “Everyone’s pretty judgmental, and, […] I felt ashamed and embarrassed to be like ‘Oh yeah, I’m a prostitute. So, can you check me?’ it just felt bad saying it to somebody.” For participants like Camila (independent indoor, Calgary), there was anticipation of negative judgment followed by discrimination:

I would never tell them [health care providers] because they’re going to judge me or they’re not going to see me. They’ll book me in, and they’re probably at the back, fighting like ‘No, you take her file’; ‘no you take her file’.

A small number of the participants who faced intersecting stigmas and did not disclose their work status mentioned that the risks of doing so were not worth the additional stigmatization they anticipated, as Julieta (independent street-based, Calgary) stated: “It’s pretty bad enough that he [my doctor] knows about the drugs, never mind that [sex work] too.” Similarly, Isabella (independent indoor, Calgary) concealed her sex work involvement by assuming the identity of a “raging slut,” the stigmatization of which was preferable than that expected from disclosure of her occupation:

[W]hen I was going in every three months and getting an STD test, my GP was getting really pissed off at me. […] I just told him I was a raging slut, and it made him most upset. He’s like ‘you know this isn’t safe for your health, right’? Meanwhile I’m like ‘You don’t even know.’ So, it would’ve been nice to be able to say that [I’m a sex worker] and not have the stigma attached to it.

##### Not Necessary for Care

Thirty-seven percent (22 out of 59) of the participants reported that their decision not to disclose was, at least in part, because they did not see the disclosure of their work as being necessary for their health care. Participants like Mila (independent street-based, St. John’s) were defiant in their response, stating that “what they don’t know don’t hurt ‘em,” or as Anne (independent street-based, Calgary) bluntly noted, “It’s none of their business.”

Other participants carefully balanced health risks and disclosure risks to determine if and when disclosure would ever be needed. Here is how Alexis (managed indoor, St. John’s) put it:

I’m one hundred per cent safe. I don’t need to do anything. I’ve never had a condom break or anything so I just know that I’m 100 per cent protected, so I just didn’t feel the need to bring it up. Now, if a condom broke or something like that I would bring it up. I wouldn’t want to bring it up, but I would.

Similarly, Jacob (independent indoor, Victoria) asserted, “If I feel that I’ve been at risk I would [disclose] but I don’t particularly feel at risk.” These discourses of right to privacy and of perceptions of low-risk work underlined a strategic decision of the participants to not mention their sex work to a health provider unless deemed necessary to receive appropriate care.

##### Confidentiality Concerns

Although less prevalent than the previous two reasons for nondisclosure, issues surrounding confidentiality were brought up by 20% (12/59) of the participants. Regarding the potential for participants’ confidentiality to be breached, family physicians were of particular concern to some participants, as Jacqueline (independent indoor, Montreal) noted, “Ideal? Mmm that I could tell my primary care physician my job without him telling my parents. Even though I know it’s confidential, I don’t really trust it.” The likelihood that medical personnel might tell participants’ family members about their sex work was worrisome, as Laura (managed indoor, Calgary) said, “I would never want her [my family doctor] to say anything to my mom.” Speaking more broadly from her experience working in the health industry, Valerie (managed indoor, Victoria) recounted, “I see it all the time when things that should be confidential are not confidential when it comes to people working in health care. I don’t think they handle it well enough.” Another element of confidentiality had to do with the recording of sex work activity on participants’ medical records, which they saw as a future threat if those medical records were subpoenaed for custody cases, other legal proceedings, or even post-secondary applications. Isabella (independent indoor, Calgary) explains:

Well I have never been able to say when I go to a doctor: ‘Hi, I’m a sex worker’ because it goes on your medical record […] and I seriously contemplated medical school, but they look at your medical record. That would’ve been an issue.

#### Experiencing Costs After Disclosure

As Table 1 shows, only 16% (16/102) of the participants who had disclosed what they did for work described only scenarios where health service providers responded negatively, along with 26% (27/102) of the participants who had experienced benefits along with costs. Post-disclosure costs reported by participants included (1) being judged, (2) inappropriate care, and (3) necessity of taking action.

##### Negative Judgment

The fears of poor treatment recounted by participants who did not disclose came true for 40% of the participants who had chosen to share their occupation with a health care provider. These included situations where health providers acted in ways that were patronizing and dehumanizing:

Well because you have to tell them what you do if you’re going for all these tests, obviously. But yeah, they can look at you like you [have] ten heads and five faces and wonder why you’re doing that with your life. (Heather, independent indoor, Kitchener-Waterloo-Cambridge)

[T]hey [providers] just treat you like you are diseased. Like they don’t want to touch you. I wanted to be like, ‘quit judging me, I’m not judging you so stop judging me’. (Dana, independent indoor, Victoria)

Hannah (independent indoor, Calgary) described her experience post-disclosure as involving both stigma and discrimination, i.e., as a “combination between being judged and not receiving care.” Similarly, Ashley (independent indoor, Victoria) said she felt “tossed to the side” by her health provider after disclosure of work status. Adele (independent indoor, Montreal) communicated how sex workers became, in the eyes of many providers, “an escort first, and then…the patient second,” lamenting that health providers “cannot get past this prostitution thing, they cannot get over it.”

Judgment sometimes extended to assumptions about participants’ integrity and ability to function in normative or untainted social roles. These types of roles included being a parent or having a “straight” job and the judgment held by providers reflected the master status of sex work, even when it was not the most prominent component of the workers’ own identity:

And telling her [the nurse] I was a sex worker she answers: ‘Ah, I understand why you’re getting an abortion.’ It’s like: ‘Hey, I could be a very good mother anyway, I’m not getting an abortion because I’m a sex worker here. That’s just my job!’ (Brigitte, managed indoor, Montreal)

I went to a therapist […] ‘Okay, this time I’m going to be totally honest, like I have to be honest if I’m going to get results.’ I told her that [I’m a sex worker] and that I wanted to go into criminology and she was just like, ‘You can’t go into criminology.’ That’s what I get for being honest, right! I couldn’t believe she was just being so blatant, so rude. (Emma, independent indoor, Calgary)

Participants reacted to providers’ disparaging treatment with stupefaction or a sense of disbelief, often followed by anger, hurt, betrayal, frustration, disillusionment, and/or regret. Shelby’s (independent indoor, Kitchener-Waterloo-Cambridge) account was typical: “She [the doctor] was being really judgemental and I felt like really disempowered. She’s a health care professional, like, it’s her job to like, just, treat her clients.”

##### Inappropriate Care

In the experience of 11% of workers who had disclosed, the health care providers had allowed their preconceived notions about sex work guide the type of health care that the sex worker was receiving. This was exemplified in Vivienne’s (independent indoor, Montreal) experience of seeking health care for an issue unrelated to sex work, but having the provider focus on sex work-related concerns:

He [the doctor] said something really weird to me when he got my STI results back, and he was like ‘oh so it’s good you don’t have HIV or syphilis’ and I was like ‘oh good’ but, because I always use protection, I didn’t see that as a risk. ‘Oh aren’t you concerned about that because you’re a sex worker?’ and I was like ‘uh….no…nope. Thanks for letting me know, it’s always good to get an STI check-up, but those were not my concerns. I came to see you for other health concerns.’

For Claire (independent indoor, Calgary), the inability to find a health care provider with whom they could speak freely about their sex work after being judged in a previous encounter resulted in the need to take on the responsibility of researching health care concerns themselves: “Well, I found that because I couldn’t find it [non-judgmental care], I ended up doing a lot of the research on my own, so I spend countless months of hours looking at STDs, photos, worst case, best case, signs, symptoms, causes, transferable rate.”

##### Necessity of Taking Action

Thirty-nine percent of the participants (40/102) described how experiencing costs associated with disclosure lead them to change their health-seeking behaviors in order to minimize costs and maximize benefits. Brienne (managed indoor, Victoria) said she keeps her sex work secret in order to keep her regular doctor but got the work-related care she needed by disclosing elsewhere:

My doctor doesn’t know what I do because she’d drop me as a patient if she did and it’s really hard to get a family doctor here. So I get testing through her once a year as my physical check-up. And I don’t, I just say ‘yeah let’s just do the whole check-up’ and I don’t tell her why. Then once a month I go to the sexual health [clinic]. They’re really great and if I have any questions about anything, they’re really, really good. And I can be really open with them about what I do.

In rare cases, the costs of disclosure necessitated participants directly challenging the attitudes of health providers in order to address the possibility of experiencing further costs in future interactions. Ashley’s (independent, indoor, Victoria) account captures the potential benefit from such an approach:

I have told him [my doctor] that I felt that he doesn’t care about me as much as he did when I was all acceptable and above board. So I think that kind of offended him and made him stand up a little straighter and I think he’s more aware [now].

Yet despite a few accounts of successful self-advocacy and education on the part of the patient, selective disclosure or concealment was the main mechanism participants employed to avoid potential stigmatization.

#### Benefiting from Disclosure

As noted in the introduction, there is a scarcity of research relating any benefits from the act of “coming out” in health care settings for individuals engaged in sex work (Abel, 2014; Nguyen et al., 2008). For the participants in our study, however, benefiting from the disclosure of work status to health providers was a dominant subtheme (see Table 1) as 57% (58/102) who had disclosed reported only experiencing benefits from sharing this information, while a further 26% (27/102) had experienced a mixture of benefits and costs. Post-disclosure benefits discussed by participants included (1) nonjudgment and relationship building and (2) targeted and appropriate care.

#### Nonjudgment and Relationship Building

For 65% of the participants (66/102), post-disclosure benefits were related to the improved interpersonal quality between patient and provider. Elizabeth (managed indoor, Victoria) was among those who had no negative experiences post-disclosure to report:

All the times that I’ve gone to a doctor, whether it be for a STD or stated STI check, whether they’ve asked me if I work in the sex industry, or if I’ve provided that information voluntarily, I’ve never been met with any kind of negative reaction.

Some participants described a low key response from health providers post-disclosure of occupation. This included Tara’s (independent indoor, Calgary) doctor who appeared not to be bothered, confirmed her regular access to STI testing, and then “moved on” or Brittany (managed indoor, Calgary) who said her doctor “tells you the risks and kind of leaves it alone.”

For some participants, such as Paige (independent indoor, Calgary), medical professionals showed a welcome willingness to listen and not to lecture; ease, openness, and nonrushed encounters contributed relationship building benefits to disclosure: “Just to picture him now: He leans up against the bed, like when I’m sitting in the chair. He’ll lean up against the bed and he just listens to me. He just looks at me and allows me to talk.” Similarly, Jennifer (independent indoor, St. John’s) described a trusting partnership with her doctor: “Like we had a great understanding towards each other and it was a good doctor relation. It was great.” This type of relationship building was also evident in the account of Kaitlyn (independent street-based, St. John’s), who stated that her doctor will “crack a joke about it [sex work], you know. He knows he can do that with me and I’m not going to get mad […] he takes care of me on my own terms.”

As noted above, for participants contending with multiple discreditable statuses, finding nonjudgmental health care was a major challenge post-disclosure. As Jade (independent street-based, Kitchener-Waterloo-Cambridge) noted, “very seldom have I felt, you know, somebody who’s empathetic towards my situation as a sex worker and substance user.” She went on to say she had found a “few providers” who showed a “genuine concern about where I was going when I left there and who I had to help me; and then trying to take steps to link me with outside sources, if I didn’t have them.” The providers that treated Jade with respect and compassion were able to become an important source of aid and resources in her health care.

##### Targeted and Appropriate Care

Thirty-nine percent of the participants (40/102) described more conspicuous gains in the care offered, often related to more targeted care that better fit their personal needs. Leila (independent indoor, Victoria) mentioned her experience was “really good” and further explains:

In fact, the last doctor I saw I was quite impressed because he was instantly like: ‘Thank you for telling me. So what we should do then is a throat swab and see if your throat has gonorrhoea.’ At the same time, he did not admonish me at all about not using condoms for oral sex, which I thought was great, to tell you the truth.

Brittany (managed indoor, Calgary) noted this type of targeted testing as well, stating: “I mean, he [the doctor] tells you ‘This is what you’re at risk for with this, this, this service’ and then just makes sure you get tested for them if you provide those services.” In the same vein, Tara (independent indoor, Calgary) related the respectful response of her family doctor after she had disclosed, “‘Oh, okay then, are you getting tested monthly?’ and I said ‘Yeah, I usually go down to the clinic.’ He says, ‘fantastic, let me know if you need testing’ and moved on.” Dana (independent indoor, Victoria) further described how her health providers made special efforts to meet her health needs:

It’s just that they [nurses] are super friendly [at the clinic]… You know I tell them; they know exactly what I do. When I come out from my testing they have a bag ready for me with condoms and lube and dental dams… I told him [the male nurse] I’m actually allergic to latex and I think three months later I went in and they had gotten latex free [condoms].

Carley (independent indoor, Victoria) described how:

when I was in the sex trade the first time, the first thing I did was I marched myself down to my doctor’s office and sat down with him and said to him ‘look, this is what I’m gonna do. Let’s have the talk’. And four years ago, I waltz myself back in and I said ‘let’s have the talk again. Guess where I’m going?’ And he is such a good guy. […] He wasn’t judgmental, he wasn’t anything. No he was really good. He really, he said ‘well, you know just make sure that you do this and do that’. I can ask him anything. I can ask him questions from clients who asked me questions and he’ll answer.

## Discussion

The depiction of occupational disclosure in health care settings has been rather stark in the sex work literature, highlighting gaps in health care access for workers compared to other citizens around the world (Aral et al., 2003; Foley, 2017; Ghimire et al., 2011; Gorry et al., 2010; Ngo et al., 2007; Phrasisombath et al., 2012; Porras et al., 2008; Scorgie et al., 2013; Stadler & Delaney, 2006). This has also been reported in Canada, where studies show that stigma is a major barrier for sex workers when accessing health care services (Bungay et al., 2013; Lazarus, Deering, Nabess, Gibson, Tyndall, & Shannon, 2012).

Like the “conditional disclosure” described by Gronholm et al. (2016) when studying young people at risk of psychosis, a significant minority of the participants in our study stated they had either chosen not to disclose their work status due to the perceived risks to negative judgment of such a decision or had experienced poor quality of care after revealing what they do for a living to a health provider. A very small minority also mentioned stigmatization based on substance use and minority gender, but these attributes did not dominate their discourses. This is likely not due to a lack of intersectional stigmatization experienced by our participants (Hankivsky et al., 2010; Earnshaw et al., 2015; Hankivsky & Christoffersen, 2008; Wailoo, 2006), but rather the nature of the question posed to participants which asked them to recall accessing health care for work-related health concerns, thereby directing them to consider their experiences in relation to sex work rather than other personal attributes. Future research to examine how intersectionality of stigmas related to race, less normative substance use, minority status, HIV, and other conditions as they play out in the health care encounters of sex workers is therefore necessary (Biradavolu et al., 2012; Ganju & Saggurti, 2017; Hatzenbuehler, Bellatorre, Lee, Finch, Muennig, & Fiscella, 2014; Surratt, O’Grady, Kurtz, Buttram, & Levi-Minzi, 2014).

Despite stories of being negatively judged and discriminated against while seeking health care, a positive finding of our study is that the majority of the participants had told a health care provider that they were sex workers and most reported they had benefited from this admission. Participants said they appreciated feeling accepted and supported, or at the very least that their health provider refrained from allowing negative judgments or personal biases to interfere with professional care. The second largest group of the participants had both positive and negative experiences with disclosure, driving many to seek, sometimes by word of mouth and sometimes by trial and error, health care provision that was both clinically appropriate and emotionally safe.

As noted above, with the exception of Abel (2014) and Nguyen et al. (2008), studies on the topic of the costs and benefits of disclosing their occupational status to health providers identify few if any benefits of “coming out” in health care settings for individuals engaged in sex work in regard to improved health care access. Our findings instead show that, similar to other stigmatized populations, adults engaged in sex work in Canada can and do gain from conditional disclosure or strategic outness (Corrigan & Matthews, 2003; Corrigan et al., 2013; Day & Schoenrade, 1997; Fisher & Akman, 2002; Gronholm et al., 2016). Sex workers described several types of benefits post-disclosure related to their health care, including increased trust and honesty within the patient-provider relationship and care that is targeted to reduce their work-related health risks. These examples of increased social support from health care providers following disclosure mirror the findings of Chaudoir and Fisher (2010) and Ragins (2008), who argue that such support is a critical mechanism that facilitates positive outcomes for individual in danger of being stigmatized, encourages the likelihood of divulging again in the future, and perpetuates a feedback loop that increases the possibility of receiving health care gains in later encounters. As health care providers are not necessarily members of the stigmatized group, receiving support from them post-disclosure “contains unique elements of affirmation and acceptance” (Ragins, 2008, p. 204) that is critical to relationship building and fostering trust. Our findings support the relevance of these arguments in the experience of revealing sex work status to health care providers and the associated benefits that many of our participants related.

A further contribution of our study is that it highlights the agency of many individuals who sell sexual services when navigating the health care bureaucracy and weighing the pros and cons of revealing their occupation (Abel, 2014; Nguyen et al., 2008). Paralleling Chaudoir and Fisher’s (2010) attention to individuals’ motives and assessments in the disclosure process, we found that sex workers’ evaluation of the potential health risks from either being denied care post-disclosure or from not being offered the most appropriate care with nondisclosure was particularly important to revealing occupation status in health care settings. Among participants who chose not to do so, many said they would not disclose unless they identified a genuine health care need (based on their own risk assessment) for a health provider to know about their sex work. By contrast, in the disclosure group, participants selectively revealed their work status to health care providers they expected to be nonjudgmental. Even when faced with costs post-disclosure, our participants were strategic health consumers, using the knowledge they gained from their health encounters to reshape their future disclosure patterns to minimize the likelihood of experiencing such costs again.

The high importance placed on the perspectives and actions of health care providers, which is so strongly evident in this study as well as our previous research on unmet health care needs (Benoit et al., 2016), lends weight to the potential effectiveness of patient-informed education for health care providers in weakening the connection between stigma and disparities in health access, health outcomes, and future health care seeking for marginalized groups (Bodkin, Delahunty-Pike, & O’Shea, 2015; Chaudoir, Earnshaw, & Andel, 2013). Gorry et al. (2010) argue prostitution stigma and other related stigmas will only be reduced when health providers understand and acknowledge the psychological burden of negative judgment on their patients, i.e., when they understand that stigma is a fundamental determinant of health (Link & Phelan, 1995; Link & Phelan, 2014) and major barrier to health equity (Pauly et al., 2009; Ruger, 2011; Sen & Östlin, 2007). Metzl and Hansen (2014) further contend that in order to combat stigma, providers must develop “structural competency,” a concept which includes recognition of the “assumptions embedded in language and attitude that serve as rhetorical social conduits for some groups of persons, and as barriers to others” (p. 128), to remove barriers to care. Our findings indicate that while many providers appear to demonstrate structural competency when providing health care to those selling sexual services, such proficiency is not guaranteed or uniformly experienced. Medical, nursing, and other health provider education programs should provide evidence-based training on the demographic characteristics of marginalized and stigmatized groups, including sex workers, research on the barriers they face when seeking health care, and tested strategies to reduce negative judgments of others whose behaviors and work activities challenge providers’ personal values and beliefs. Health care providers should also receive information in their formal training about “outreach organisations and social services that offer forms of support for sex workers and their relationships with the police, planners and other bodies” (Laing & Cook, 2014, p. 512). Involving sex workers in the development of such training modules is fundamental to their success in the classroom, health clinics, and related practice settings. Sex workers are members of local communities and health care providers need to also become knowledgeable about how to help workers navigate available systems of care so that they can avoid potentially stigmatizing situations (Benoit, Belle-Isle, Smith, Phillips, Shumka, Atchison, Jansson et al., 2017). Future studies should investigate this navigation process and discover how to better enable sex workers to shape their own personal practices around health promotion and prevention strategies, and to contribute to improving their access to health and social services within the local community.

## Strengths and Limitations

The findings described above should be evaluated within our study’s limitations. Our nonrandom sample is not statistically representative of the hard-to-reach population of individuals engaged in sex work in Canada (Benoit et al., 2005). The study is also limited due to nonparticipation by those who did not want to discuss their sex work with interviewers, were concerned about their confidentiality, or were prevented from participating either by a person or due to lack of time or inclination. Having only recruited participants in six urban areas of Canada, important barriers to disclosure of work status that were pertinent in other municipalities or rural regions in the country at the time of data collection may have been missed.

Despite these limitations, this is the only qualitative study of which we are aware that captures the health-seeking experiences of a relatively large and diverse sample of adults engaged in sex work. In the end, we feel that our sample reflects the diversity of adults involved in selling sexual services in Canada and captured rich qualitative data on a range of issues relevant to sex workers’ health, safety, and social rights. This may contribute to why the findings describe a more positive view of disclosure of work status in health encounters than has previously been reported from research with more limited homogeneous samples from one geographical location.

## Conclusions

Overall, we found convincing evidence for a connection between sex workers’ unmet health needs and the agency they demonstrate in navigating health care encounters in order to meet their health needs. Discussions of disclosure or nondisclosure, judgment or nonjudgment, and discrimination or caring characterize their health care-seeking experiences. People who sell sexual services, like other health users, are voicing that trust in the patient-provider relationship is not a given, but rather continuously won in practice. Increased trust leads to disclosure of one’s work background, which creates an opportunity for health care providers to appropriately serve patients and ensure they are getting the information, supplies, and tests they require. Health professionals, as well as other service professionals and policy makers, need to learn new ways to converse with and learn from people in sex work jobs that take into account the heavy costs associated with prostitution stigma (Benoit et al., 2017; Sanders, 2017) and continue to develop “structural competency” when dealing with populations such as sex workers who may be at a disadvantage for having their health care needs met in a safe and appropriate manner. Structural competency would go some way in addressing the formidable health inequities some sex workers face when accessing the needed care.
